# Prevalence and main factors influencing anxiety symptoms in Chinese older adults with cataracts: a national cross-sectional survey

**DOI:** 10.3389/fpsyt.2025.1534863

**Published:** 2025-06-02

**Authors:** Fadong Zhang, Xiang Li, Ketong Liu, Lanzhi Xie, Lu Wan, Shiwei Cao, Yandi Fu, Tengfei Niu

**Affiliations:** ^1^ Nursing Department, People’s Hospital of Xiushan County, Chongqing, China; ^2^ The First Clinical College, Chongqing Medical University, Chongqing, China; ^3^ The Fifth Clinical College, Chongqing Medical University, Chongqing, China; ^4^ The Second Clinical College, Chongqing Medical University, Chongqing, China; ^5^ School of Pediatrics, Chongqing Medical University, Chongqing, China; ^6^ Department of Basic Courses, Chongqing Medical and Pharmaceutical College, Chongqing, China

**Keywords:** anxiety symptoms, cataracts, influencing factors, random forest, mental health

## Abstract

**Background:**

The mental health of cataract patients should not be ignored; however, little is known from existing studies about anxiety symptoms in Chinese older adults with cataracts. The aim of this study was to assess the prevalence of anxiety symptoms and their key influencing factors among Chinese older adults with cataracts.

**Methods:**

Based on the cross-sectional data published in 2018 by the Chinese Longitudinal Healthy Longevity Survey (CLHLS), 1,320 cataract patients aged 65 and older were finally included. Anxiety symptoms were assessed using the Generalised Anxiety Disorder (GAD-7) scale. Logistic regression analyses were performed on 24 variables from three dimensions: socio-demographic characteristics, health status and lifestyle, in order to investigate the influencing factors of anxiety symptoms in older adults with cataracts. Meanwhile, this study developed an advanced Random Forest Model to rank the importance of the significant influencing factors in the logistic regression, providing an in-depth understanding for further precision prevention and management.

**Results:**

The prevalence of anxiety symptom in Chinese older adults with cataracts was 11.06%. Logistic regression analyses showed that poor economic status (OR=3.162, 95% CI:1.719-5.817), obesity(Body Mass Index>28) (OR=2.11,95%CI:1.10-4.05), poor self-reported health (OR=1.91,95%CI:1.11-3.30), sleeping less than 7h(OR=1.98,95%CI:1.30-3.01), having fair (OR=1.61,95%CI:1.03-2.53) or poor (OR=2.70,95%CI:1.21-6.02) life satisfaction, and having hearing impairment (OR=1.72,95%CI:1.12-2.65) were risk factors for anxiety symptoms in cataract patients, eating fresh fruits (OR=0.587, 95%CI:0.369-0.933) and age (OR=0.97,95%CI:0.94-0.99) were protective factors for anxiety symptoms in cataract patients. The results of Random Forest showed that age was the most important factor affecting anxiety symptoms in cataract patients, followed by Body Mass Index, self-reported health, economic status, eating fruits or not, sleep duration, life satisfaction, and hearing impairment.

**Conclusion:**

The results of the study showed that the anxiety symptoms of cataract patients should not be ignored and are influenced by a variety of factors. Healthcare workers should actively conduct mental health screening and strengthen the monitoring of important influencing factors in the treatment and care of cataract patients.

## 1 Introduction

Cataract is a chronic eye disease that causes vision loss due to clouding of the lens (1).It is also one of the leading causes of preventable blindness.According to the statistics of 2023, the total number of cataract cases in China had exceeded 18 million from 1990 to 2019, and it was projected that the number of cataract cases in China would rise to 43 million by 2030 and the prevalence of cataract in the 45–89 age group would reach 33.34% (2). In addition to causing severe disability and dysfunction, cataract is associated with an increased risk of psychological problems such as depressive symptoms, anxiety symptoms (3–5). In a 2019 study, Ann IY et al. showed that anxiety in cataract patients exacerbated insomnia symptoms in cataract patients (6). This may further delay the recovery of cataract patients after surgery and increase their mortality. Results from another study suggested that anxiety symptoms may lead to increased intraocular pressure or blood pressure in cataract patients undergoing cataract surgery (5). This may cause patients to experience more severe pain and pose a significant impediment to cataract treatment and prognosis (7). Therefore, a better understanding of the clinical epidemiological characteristics of anxiety symptoms among cataract patients would help in the early identification of those at high risk of anxiety symptoms, thereby improving patients’ physical and mental health.

Anxiety disorders are common neurological disorders that consist of three main types: trait anxiety, state anxiety, and social anxiety, which are generally characterised by excessive worry, fear, and apprehension, and are usually accompanied by specific somatic symptoms, such as sweating, dizziness, and shortness of breath or even insomnia, irritability, and muscle aches and pains (8, 9).Anxiety symptoms are highly persistent, usually manifest as chronic episodes, and often co-exist with other psychiatric disorders (10). It has been reported that approximately 4.7% of older adults in China suffer from anxiety symptoms (11). Long-term anxiety symptoms may lead to a range of health problems, such as easy fatigue and poor immunity, which can further develop into chronic illnesses, such as heart disease, stroke and other chronic condition (10). Additionally,there is also strong association between anxiety symptoms and higher rates of suicide and mortality (12). The development of anxiety symptoms is primarily linked to physical risk factors (e.g., cognitive impairment, high blood pressure, poor health self-assessment, and impaired vision or hearing), psychological risk factors (e.g., lack of self-efficacy, history of mental illness, and dysfunction), and social risk factors (e.g., lack of social support, overprotection during childhood, and low income) (13).Various factors may ultimately activate neuroinflammatory pathways, leading to neuronal pathway and neurotransmitter dysfunction i.e., reduction of serotonin, dopamine, and norepinephrine in the synaptic gap, which ultimately leads to the development of anxiety disorders (14, 15). Numerous previous studies have reported on the factors influencing anxiety symptoms in older adults. He, ZF et al. showed that being female, being widowed, and having a chronic disease were risk factors for anxiety symptoms in Chinese older adults, whereas living in a rural area and walking every day were protective factors for anxiety symptoms in older adults (16). Other studies have focused on possible measures for preventing anxiety symptoms in older adults from the perspectives of lifestyle and health status (17, 18). In addition, some studies have also reported on anxiety and depression in older adults with visual impairment, and the results suggest that relatively young age, depressive symptoms, not living alone, and working difficultly are risk factors for anxiety symptoms in older adults with visual impairment (19). Undoubtedly, the results of all of these studies have played a prominent role in preventing and ameliorating anxiety symptoms in older adults in recent years; nonetheless, the results of these studies may still be inapplicable in Chinese older adults with visual impairment, especially cataract patients. As the mental health of Chinese cataract patients remains understudied, it is important to understand the prevalence of anxiety symptoms and its main influencing factors in Chinese cataract patients in order to address this gap.

In terms of research methodology, previous studies related to cataract tend to apply traditional logistic regression analysis. As a machine learning algorithm, Random Forest(RF) is an excellent research tool because of its powerful classification ability and easy-to-interpret learning mechanism. In recent years, Random Forest has been widely used in the medical field for diagnosis and classification of disease (20), prediction of clinical outcomes (21), and estimation of the significance of exposure to pathogenic factors (22). RF is an integrated learning method based on decision trees, and by analysing the splitting points of each decision tree, we can visually understand how the model makes predictions (23). Other machine learning models such as XGBoost (Extreme Gradient Boosting) can provide better prediction accuracy, but their interpretability is relatively poor, especially when dealing with high-dimensional data, the internal mechanism of the model is more complex (24). Black-box models, such as neural networks, are also difficult to explain their internal decision-making process (25). In addition, RF is able to handle missing values, noise and small sample sizes well when the data volume is moderate (23). It requires less data preprocessing and can handle high-dimensional features, which makes it suitable for many practical problems. In the application scenario of medical research, the stability, reliability and interpretability of the model may be more important than the mere prediction accuracy. Therefore, this study proposes to combine Logistic Regression with Random Forest, and further use Random Forest Model to rank the importance of significant influencing factors in Logistic Regression. The application of this more novel approach to data analysis may help to gain a deeper understanding of the causes of anxiety symptoms in cataract patients and to intervene and manage them with precision.

In summary, given the clinical importance of anxiety symptoms among cataract patients and the limitations in previous studies, we used the national cross-sectional data published in 2018 by the Chinese Longitudinal Healthy Longevity Survey (CLHLS) to analyze the influencing factors of anxiety symptoms in older adults with cataracts in China from multiple perspectives, and measured the importance of these factors through a Random Forest Model. It has important reference value for alleviating anxiety symptoms and promoting the overall mental health of older adults with cataracts.

## 2 Materials and methods

### 2.1 Study population

The data for this study came from the Chinese Longitudinal Healthy Longevity Survey(CLHLS) organized by the Centre for Healthy Aging and Development at Peking University,which is a nationally representative prospective cohort survey of older people aged 65 years and over.It used a multi-stage, disproportionate and targeted random sampling method (26), covering 631 cities in 23 provinces of China, and included surveys on socio-demographic characteristics, social conditions, economic conditions, and health conditions. The CLHLS project was approved by the Biomedical Ethics Committee of Peking University (IRB00001052-13074). All participants or their proxy respondents provided informed consent (27).

**Figure 1:**
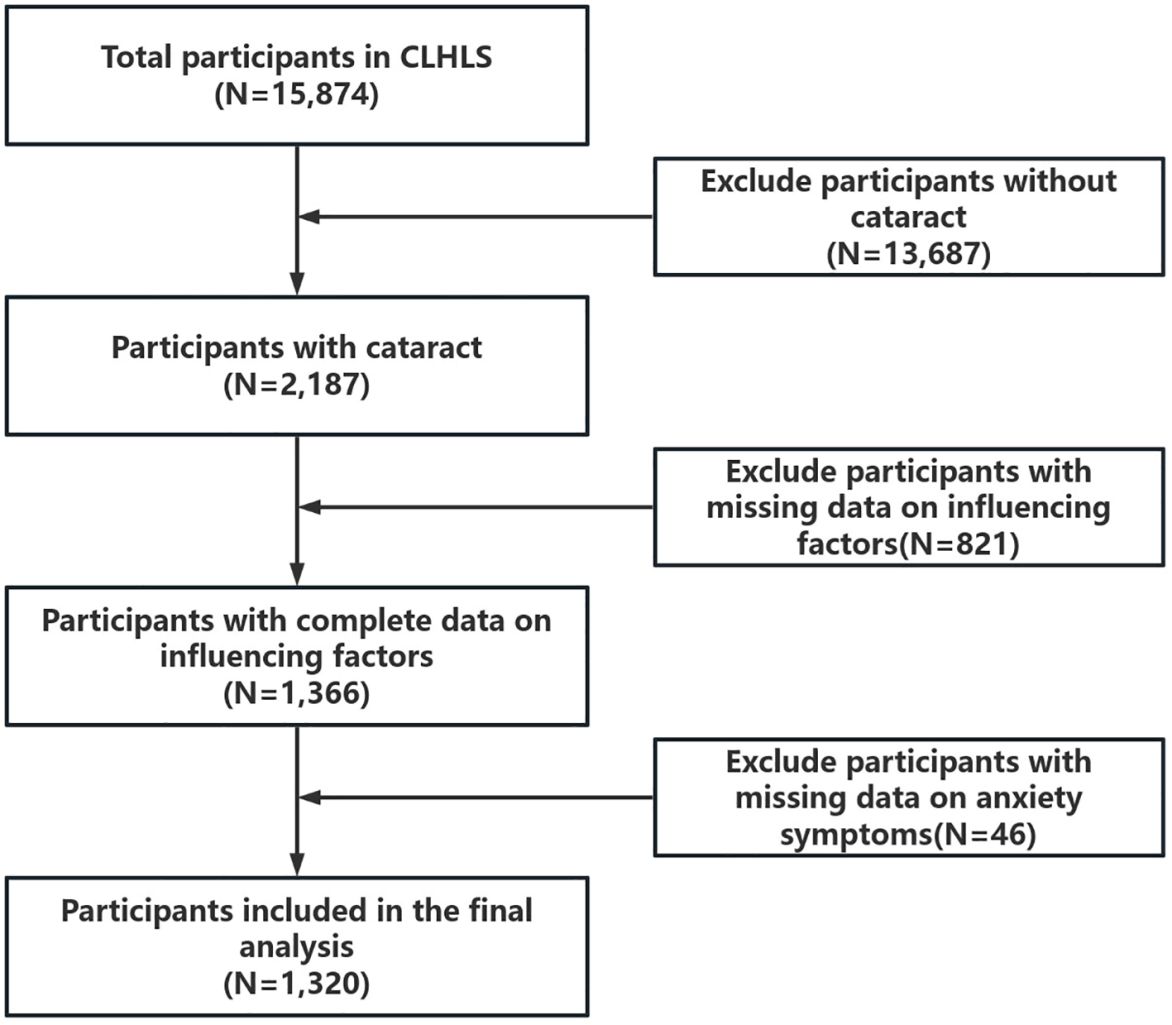
Flowchart of the inclusion of participants, showing the detailed data-cleaning process.

This study used data published by CLHLS in 2018.The selection of the study sample was based on the question in the questionnaire, ‘Are you currently diagnosed with cataracts by hospital?’. Respondents who answered ‘yes’ to this question were included in our study. There were 15,874 participants in CLHLS in 2018, of which 2,187 participants were diagnosed with cataract by a clinician at the hospital. Participants who lacked information on anxiety symptoms and related influencing factors were further excluded to ensure accuracy and reliability of data analysis and to reduce selection biases. The study ultimately included 1,320 participants aged 65 years or older. The detailed data cleaning process is shown in **Figure 1**.

### 2.2 Outcome variables

Anxiety symptoms in older adults were assessed using the Generalised Anxiety Disorder (GAD-7) scale (28). The GAD-7 scale consists of seven questions, including “feeling uneasy, worried, and annoyed”, “can’t stop or control worrying”, “worrying too much about things”, “very nervous and difficult to relax”, “very anxious and therefore unable to sit still”, “becoming easily annoyed”, “feeling that something terrible has happened”.Each question was scored on a scale ranging from 0 to 3 (0 = never, 1 = few days, 2 = more than half of the days, 3 = almost every day), and the total score for the seven questions ranged from 0 to 21, with a total score on the scale of greater than or equal to 5 considered to be suffering from anxiety symptoms (28). The results of previous studies have demonstrated the utility of the scale in the Chinese older adult population (29). Cronbach’s α of GAD-7 in this study was 0.914.

### 2.3 Influencing factors

Based on the availability of data resources in the CLHLS database and the exploration of factors influencing anxiety symptoms in Chinese older adults in previous studies (22, 30, 31), we sorted out 24 influencing factors that may affect the anxiety symptoms of cataract patients at 3 levels, namely, socio-demographic characteristics, health status, and lifestyle.

Socio-demographic characteristics included age, gender, education, marital status, place of residence, and economic status.

Health status included Body Mass Index, abdominal obesity, hearing impairment, hypertension, diabetes, cardiopathy, self-reported health, and Activities of Daily Living disability.

Lifestyle included eating vegetables and fruits, taste, sleep duration, smoking, drinking, exercise, physical labour, social activity, and life satisfaction.

Detailed variable measurements and variable assignments are shown in **Appendix I**.

### 2.4 Statistical analyses

The samples were used for descriptive statistical analysis after data cleaning. Kolmogorov-Smirnov test was used to determine normal distribution of continuous variables. Continuous variables conforming to normal distribution were expressed as mean ± standard deviation (M ± SD) and categorical variables were expressed as frequency and percentage (n (%)).Two independent samples t-test and chi-square test were used for between-group comparisons. Bonferroni correction was used to rule out the problem of multiple comparisons between variables and to avoid false-positive results. Binary logistic regression was adopted to explore the factors influencing the anxiety symptoms of cataract patients, and variance inflation factor (VIF) was used to detect possible collinearity problems between variables in the regression model, and VIF<10 was considered to be free of collinearity problems.

In order to assess the significance of different influencing factors, we ranked the random forest importance of statistically significant variables in the logistic regression model. Based on the statement of Transparent Reporting of a Multivariable Prediction Model for Individual Prognosis or Diagnosis(TRIPOD), this study was conducted by randomly selecting 70% of the overall data as the training set and 30% as the test set (32). This division criterion ensured that the model had enough data for effective training, and also ensured the representativeness of the test set to avoid overfitting or underfitting of the model, which demonstrated good utility in model construction in previous medical studies (33). The optimal parameters of the model were obtained using grid optimisation, the model was tested by the 10-fold cross-validation method, and and a random forest model with parameters mtry of 3 and ntree of 500 was finally built. Compared with traditional regression methods, the random forest algorithm can avoid increasing estimated parameters and being sensitive to missing values or outliers when dealing with multilevel categorical variables, so the application of RF reduces bias, tolerates outliers, and reduces overfitting of the model, resulting in more accurate and stable predictions for the study (34).The final order of importance of the influencing factors, as reflected in the average reduction of the Gini index, is obtained. Specifically, the greater the average decrease in the Gini index for a variable, the more important that variable is (35). The performance of the model was assessed using Area Under Curve (AUC), accuracy, sensitivity, precision, recall, and F1 score. All data analyses were performed using SPSS 26.0 and R 4.3.0 and P<0.05 (two-sided) was considered statistically significant.

## 3 Results

### 3.1 Descriptive analysis of the sample

#### 3.1.1 Socio-demographic characteristics

A total of 1,320 cataract patients were included in this study, of which 146 had anxiety symptoms with a prevalence of 11.06%. The mean age of the respondents was 85.30 ± 10.50. About 43.33% were males, 67.42% lived in urban areas, and 40.15% of the respondents had less than 6 years of education. Only 8.26% of the respondents were dissatisfied with their economic status. The t-test results showed that in the group of cataract patients, the mean age of patients with anxiety symptoms was lower than that of patients without anxiety symptoms (p=0.013). The results of chi-square test showed that there was a statistically significant difference in the prevalence of anxiety symptoms among cataract patients across years of education and economic status (p<0.05), and the detailed results were displayed in **Table 1**.

**Table 1:** Basic description and differential analysis of socio-demographic characteristics.

Variables	Total samples	Normal samples	Anxiety symptoms	Statistic	P
All participants,n(%)	1,320	1,174 (88.94)	146 (11.06)		
Age, Mean ± SD	85.30 ± 10.50	85.55 ± 10.49	83.27 ± 10.34	t=2.48	0.013
Gender, n (%)				χ²=1.66	0.198
Female	748 (56.67)	658 (56.05)	90 (61.64)		
Male	572 (43.33)	516 (43.95)	56 (38.36)		
Education years, n (%)				χ²=8.58	0.014
0	530 (40.15)	458 (39.01)	72 (49.32)		
0-6	401 (30.38)	356 (30.32)	45 (30.82)		
>6	389 (29.47)	360 (30.66)	29 (19.86)		
Marital status, n (%)				χ²=2.67	0.102
Not married	779 (59.02)	702 (59.80)	77 (52.74)		
Married	541 (40.98)	472 (40.20)	69 (47.26)		
Place of residence, n (%)				χ²=2.50	0.114
Rural	430 (32.58)	374 (31.86)	56 (38.36)		
Urban	890 (67.42)	800 (68.14)	90 (61.64)		
Economic status, n (%)				χ²=55.69	<.001
Good	329 (24.92)	306 (26.06)	23 (15.75)		
Fair	882 (66.82)	794 (67.63)	88 (60.27)		
Bad	109 (8.26)	74 (6.30)	35 (23.97)		

t, t-test; χ², Chi-square test; SD, Standard Deviation; Mean ± SD refers to participants’ age.

#### 3.1.2 Health status

In terms of health status (**Table 2**), nearly half (44.47%) of the respondents in this study reported their self-reported health as “good”. At the disease-specific level, 49.7% of the respondents had a Body Mass Index within the normal range. Abdominal obesity, hearing impairment, and cardiopathy were also present in nearly half of the respondents, at 46.67%, 42.05%, and 41.59%, respectively. Fewer respondents had diabetes (32.95%) and more respondents had hypertension(62.73%). The results of chi-square test showed that the prevalence of anxiety symptoms in cataract patients was statistically significant (p<0.05) between different hearing impairment, self-reported health.

**Table 2:** Basic description and differential analysis of health status.

Variables	Total samples	Normal samples	Anxiety symptoms	Statistic	P
BMI, n (%)				χ²=6.40	0.094
<18.5	174 (13.18)	154 (13.12)	20 (13.70)		
18.5-24	656 (49.70)	591 (50.34)	65 (44.52)		
24-28	379 (28.71)	338 (28.79)	41 (28.08)		
>28	111 (8.41)	91 (7.75)	20 (13.70)		
Abdominal obesity, n (%)				χ²=0.73	0.392
No	704 (53.33)	631 (53.75)	73 (50.00)		
Yes	616 (46.67)	543 (46.25)	73 (50.00)		
Hearing impairment, n (%)				χ²=4.26	0.039
No	765 (57.95)	692 (58.94)	73 (50.00)		
Yes	555 (42.05)	482 (41.06)	73 (50.00)		
Hypertension, n (%)				χ²=0.97	0.325
No	492 (37.27)	443 (37.73)	49 (33.56)		
Yes	828 (62.73)	731 (62.27)	97 (66.44)		
Diabetes, n (%)				χ²=0.34	0.561
No	885 (67.05)	784 (66.78)	101 (69.18)		
Yes	435 (32.95)	390 (33.22)	45 (30.82)		
Cardiopathy, n (%)				χ²=0.34	0.560
No	771 (58.41)	689 (58.69)	82 (56.16)		
Yes	549 (41.59)	485 (41.31)	64 (43.84)		
SRH, n (%)				χ²=51.40	<.001
Good	587 (44.47)	548 (46.68)	39 (26.71)		
Fair	499 (37.80)	448 (38.16)	51 (34.93)		
Bad	234 (17.73)	178 (15.16)	56 (38.36)		
ADL disability, n (%)				χ²=1.61	0.204
No	988 (74.85)	885 (75.38)	103 (70.55)		
Yes	332 (25.15)	289 (24.62)	43 (29.45)		

t, t-test; χ², Chi-square test; SD, Standard Deviation; BMI, Body Mass Index; SRH, Self-Reported Health; ADL, Activities of Daily Living.

#### 3.1.3 Lifestyle

In terms of lifestyle (**Table 3**), 31.36% of the respondents consumed fruits almost every day, 69.02% consumed vegetables almost every day, 68.64% maintained a light diet, and 62.35% slept for more than 7 hours. About 44.47% of the respondents exercised regularly, 28.48% smoked, and 23.03% drank. About 65.91% of the participants were engaged in physical labor and 68.26% experienced low social activities. Only 3.64% of the respondents rated their quality of life as “poor”. The results of the chi-square test showed a statistically significant difference (p<0.05) in the prevalence of anxiety symptoms among cataract patients between different frequency of eating fruits, frequency of eating vegetables, sleep duration, exercise, physical labor, social activities and life satisfaction.

**Table 3:** Basic description and differential analysis of lifestyle.

Variables	Total samples	Normal samples	Anxiety symptoms	Statistic	P
Eating fruits				χ²=14.04	<.001
Never or rarely	241 (18.26)	198 (16.87)	43 (29.45)		
Occasionally or quite often	665 (50.38)	599 (51.02)	66 (45.21)		
Almost everyday	414 (31.36)	377 (32.11)	37 (25.34)		
Eating vegetables				χ²=6.93	0.031
Never or rarely	39 (2.95)	34 (2.90)	5 (3.42)		
Occasionally or quite often	370 (28.03)	316 (26.92)	54 (36.99)		
Almost everyday	911 (69.02)	824 (70.19)	87 (59.59)		
Taste, n (%)				χ²=0.63	0.426
Not light	414 (31.36)	364 (31.01)	50 (34.25)		
Light	906 (68.64)	810 (68.99)	96 (65.75)		
Sleep duration, n (%)				χ²=29.76	<.001
7-9	570 (43.18)	526 (44.80)	44 (30.14)		
<7	497 (37.65)	412 (35.09)	85 (58.22)		
>9	253 (19.17)	236 (20.10)	17 (11.64)		
Smoking, n (%)				χ²=0.10	0.758
No	944 (71.52)	838 (71.38)	106 (72.60)		
Yes	376 (28.48)	336 (28.62)	40 (27.40)		
Drinking, n (%)				χ²=0.30	0.584
No	1016 (76.97)	901 (76.75)	115 (78.77)		
Yes	304 (23.03)	273 (23.25)	31 (21.23)		
Exercise, n (%)				χ²=8.93	0.003
No	733 (55.53)	635 (54.09)	98 (67.12)		
Yes	587 (44.47)	539 (45.91)	48 (32.88)		
Physical labour, n (%)				χ²=8.53	0.003
No	450 (34.09)	416 (35.43)	34 (23.29)		
Yes	870 (65.91)	758 (64.57)	112 (76.71)		
Social activity, n (%)				χ²=4.57	0.032
No	419 (31.74)	384 (32.71)	35 (23.97)		
Yes	901 (68.26)	790 (67.29)	111 (76.03)		
Life satisfaction, n (%)				χ²=52.62	<.001
Good	930 (70.45)	857 (73.00)	73 (50.00)		
Fair	342 (25.91)	287 (24.45)	55 (37.67)		
Bad	48 (3.64)	30 (2.56)	18 (12.33)		

t, t-test; χ², Chi-square test; SD, Standard Deviation.

### 3.2 Binary logistic regression results

**Figure 2:**
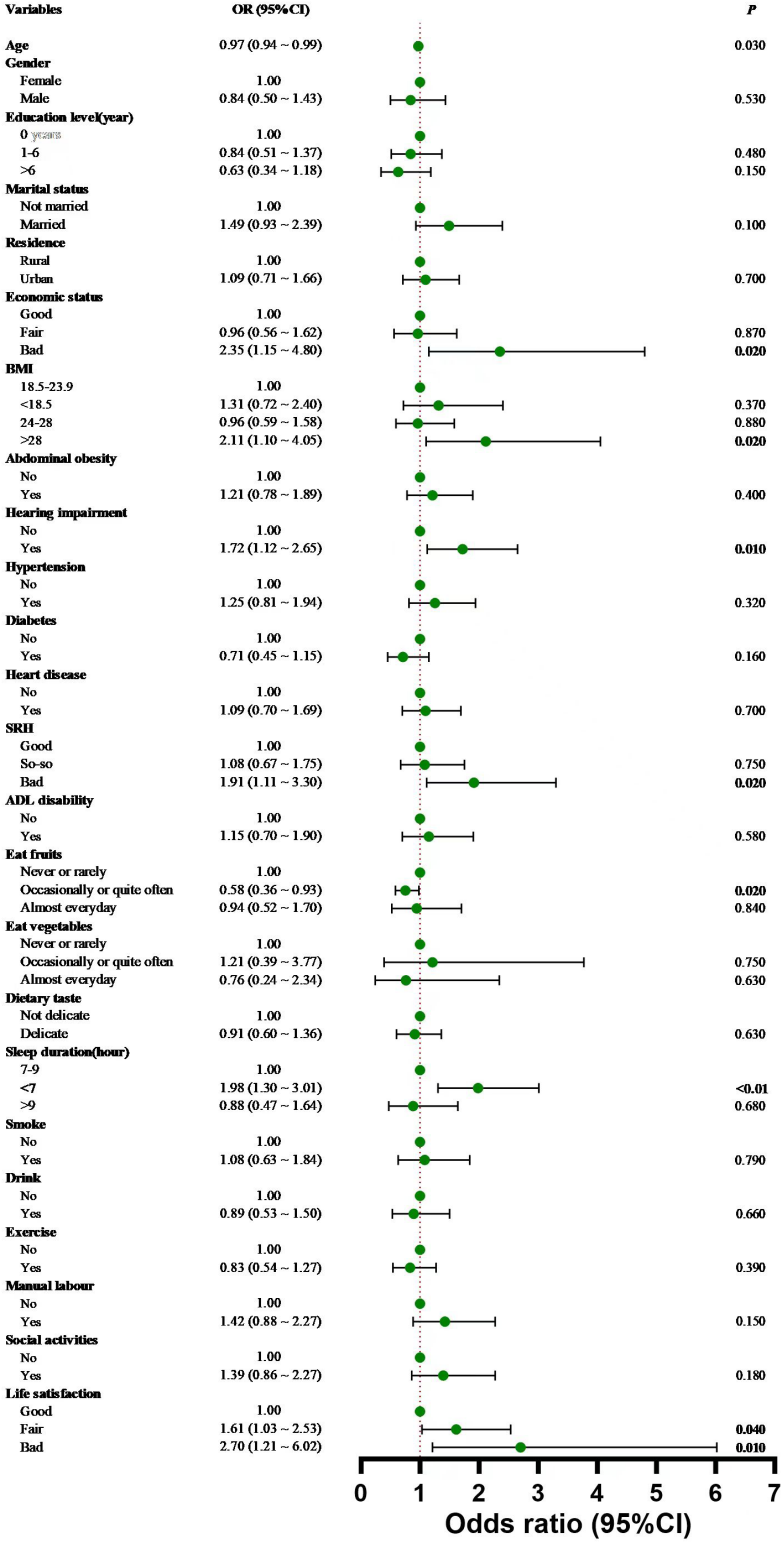
Forest plot based on the results of Binary logistic regression analysis. Error bars represent 95% confidence intervals, and dots indicate odds ratios.

Multifactional logistic regression analysis suggested that age, BMI, SRH, economic status, eating fruits, sleep duration, life satisfaction, and hearing impairment were significant influencing factors on anxiety symptoms in cataract patients (**Table 4**, **Figure 2**).

**Table 4:** Binary logistic regression analysis results.

Variables	β	S.E	Z	P	OR (95%CI)	VIF
Age	-0.03	0.01	-2.21	0.03	0.97 (0.94 ~ 0.99)	1.497
Gender						1.338
Female					1.00 (Reference)	
Male	-0.17	0.27	-0.63	0.53	0.84 (0.50 ~ 1.43)	
Education						1.171
0					1.00 (Reference)	
0-6	-0.18	0.25	-0.71	0.48	0.84 (0.51 ~ 1.37)	
>6	-0.46	0.32	-1.44	0.15	0.63 (0.34 ~ 1.18)	
Marital status						1.255
Not married					1.00 (Reference)	
Married	0.4	0.24	1.66	0.1	1.49 (0.93 ~ 2.39)	
Place of esidence						1.120
Rural					1.00 (Reference)	
Urban	0.08	0.22	0.38	0.7	1.09 (0.71 ~ 1.66)	
Economic status						1.082
Good					1.00 (Reference)	
Fair	-0.05	0.27	-0.17	0.87	0.96 (0.56 ~ 1.62)	
**Bad**	**0.85**	**0.36**	**2.34**	**0.02**	**2.35 (1.15 ~ 4.80)**	
BMI						1.079
18.5-24					1.00 (Reference)	
<18.5	0.27	0.31	0.89	0.37	1.31 (0.72 ~ 2.40)	
24-28	-0.04	0.25	-0.15	0.88	0.96 (0.59 ~ 1.58)	
**>28**	**0.75**	**0.33**	**2.26**	**0.02**	**2.11 (1.10 ~ 4.05)**	
Abdominal obesity						1.174
No					1.00 (Reference)	
Yes	0.19	0.23	0.85	0.4	1.21 (0.78 ~ 1.89)	
Hearing impairment						1.154
No					1.00 (Reference)	
**Yes**	**0.54**	**0.22**	**2.48**	**0.01**	**1.72 (1.12 ~ 2.65)**	
Hypertension						1.139
No					1.00 (Reference)	
Yes	0.22	0.22	1	0.32	1.25 (0.81 ~ 1.94)	
Diabetes						1.190
No					1.00 (Reference)	
Yes	-0.34	0.24	-1.39	0.16	0.71 (0.45 ~ 1.15)	
Cardiopathy						1.170
No					1.00 (Reference)	
Yes	0.09	0.22	0.39	0.7	1.09 (0.70 ~ 1.69)	
SRH						1.097
Good					1.00 (Reference)	
So-so	0.08	0.25	0.32	0.75	1.08 (0.67 ~ 1.75)	
**Bad**	**0.65**	**0.28**	**2.34**	**0.02**	**1.91 (1.11 ~ 3.30)**	
ADL disability						1.208
No					1.00 (Reference)	
Yes	0.14	0.26	0.56	0.58	1.15 (0.70 ~ 1.90)	
Eating fruits						1.113
Never or rarely					1.00 (Reference)	
**Occasionally or quite often**	**-0.54**	**0.24**	**-2.25**	**0.02**	**0.58 (0.36 ~ 0.93)**	
Almost everyday	-0.06	0.3	-0.21	0.84	0.94 (0.52 ~ 1.70)	
Eating vegetables						1.070
Never or rarely					1.00 (Reference)	
Occasionally or quite often	0.19	0.58	0.32	0.75	1.21 (0.39 ~ 3.77)	
Almost everyday	-0.28	0.58	-0.48	0.63	0.76 (0.24 ~ 2.34)	
Taste						1.020
Not light					1.00 (Reference)	
Light	-0.1	0.21	-0.48	0.63	0.91 (0.60 ~ 1.36)	
Sleep duration						1.044
7-9					1.00 (Reference)	
**<7**	**0.68**	**0.21**	**3.19**	**<0.01**	**1.98 (1.30 ~ 3.01)**	
>9	-0.13	0.32	-0.42	0.68	0.88 (0.47 ~ 1.64)	
Smoking						1.243
No					1.00 (Reference)	
Yes	0.07	0.27	0.27	0.79	1.08 (0.63 ~ 1.84)	
Drinking						1.144
No					1.00 (Reference)	
Yes	-0.12	0.26	-0.44	0.66	0.89 (0.53 ~ 1.50)	
Exercise						1.110
No					1.00 (Reference)	
Yes	-0.19	0.22	-0.86	0.39	0.83 (0.54 ~ 1.27)	
Physical labour						1.150
No					1.00 (Reference)	
Yes	0.35	0.24	1.44	0.15	1.42 (0.88 ~ 2.27)	
Social activity						1.184
No					1.00 (Reference)	
Yes	0.33	0.25	1.33	0.18	1.39 (0.86 ~ 2.27)	
Life satisfaction						1.114
Good					1.00 (Reference)	
**Fair**	**0.48**	**0.23**	**2.08**	**0.04**	**1.61 (1.03 ~ 2.53)**	
**Bad**	**0.99**	**0.41**	**2.43**	**0.01**	**2.70 (1.21 ~ 6.02)**	

β, standardized regression coefficient; SE, Standard Error; Z, standard score; P, P value; OR, Odds Ratio; CI, Confidence Interval; VIF, Variance Inflation Factor; BMI, Body Mass Index; SRH, Self-Reported Health; ADL, Activities of Daily Living. Bold vales highlight significant variables.

Specifically, for each additional year of age, the risk of anxiety symptoms in cataract patients decreased by 3% (OR = 0.97, 95% CI:0.94 ~ 0.99). Cataract patients with poor economic status (OR=2.35, 95%CI:1.15 ~ 4.80) had a higher risk of prevalence of anxiety symptoms compared to those with good economic status. Cataract patients who ate fruits (OR=0.58; 95%CI:0.36 ~ 0.93) were 42% less likely to develop anxiety symptoms compared to those who did not eat fruits. Cataract patients (OR=1.98, 95%CI:1.30 ~ 3.01) who slept fewer hours of sleep (<7h) had a 1.98-fold increased risk of suffering from anxiety symptoms compared to those who had normal sleep hours(7-9h). Compared with cataract patients with good life satisfaction, those with fair (OR=1.61, 95%CI:1.03 ~ 2.53) or poor (OR=2.70, 95%CI:1.21 ~ 6.02) life satisfaction had a 1.61-fold and 2.70-fold increased risk of suffering from anxiety symptoms, respectively. Cataract patients with a BMI >28 (OR=2.11, 95%CI:1.10 ~ 4.05) were 2.11 times more likely to have anxiety symptoms than those with a normal BMI (18.5-24). Cataract patients with poor self-reported health(OR=1.91, 95%CI:1.11 ~ 3.30) had a 1.91-fold increased risk of suffering from anxiety symptoms compared to those with good self-reported health. Cataract patients with hearing impairment (OR=1.72, 95% CI:1.12-2.65) had a higher risk of prevalence of anxiety symptoms compared to those without hearing impairment. There was no collinearity among all variables.

### 3.3 Random forest results

**Figure 3:**
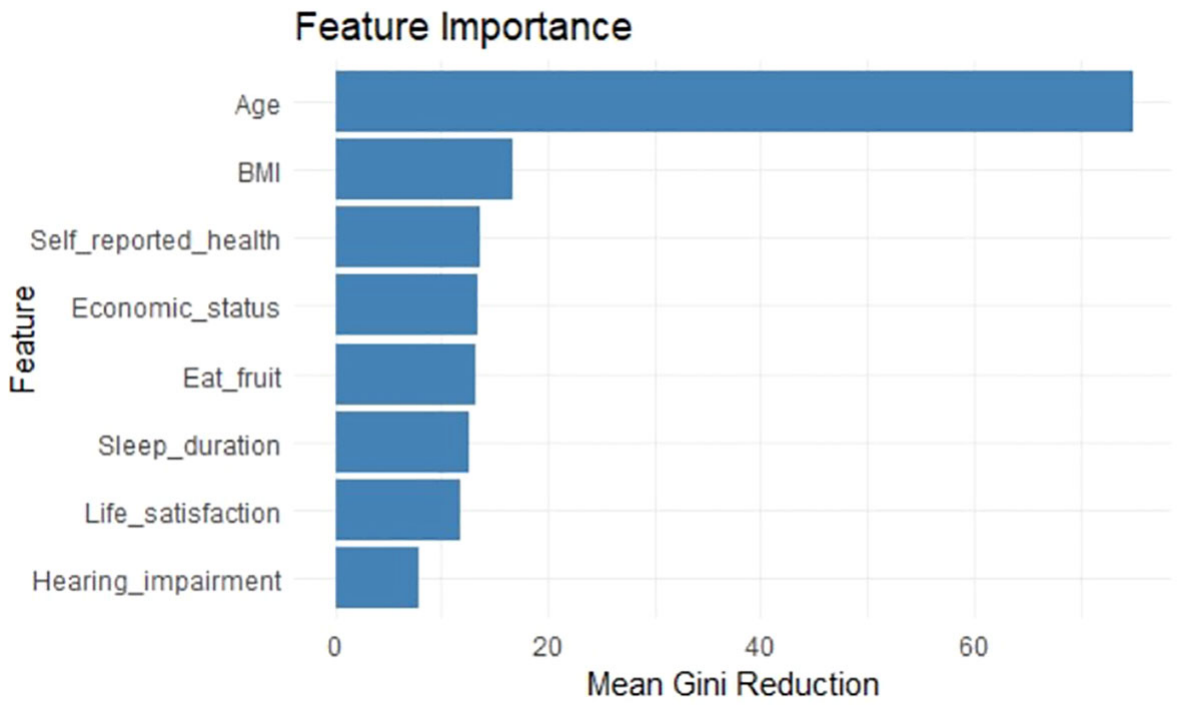
Importance ranking of variables with decreasing average Gini index based on random forest model. The bar graph represents the extent to which each variable affects the dependent variable and the rank of importance in the random forest model.

The results of the Random Forest showed that age was the most important factor affecting anxiety symptoms in cataract patients, followed by BMI, self-reported health, economic status, eating fruits or not, sleep duration, life satisfaction and hearing impairment(**Figure 3**). **Table 5** displays the performance indicators of the random forest model on the training set and the test set.

**Table 5:** Model performance evaluation metrics.

Data set	AUC	Sensitivity	Accuracy	Precision	Recall	F_1_score
Training set	0.979	0.979	0.987	0.987	0.979	0.993
Testing set	0.740	0.960	0.876	0.906	0.960	0.932

AUC, Area Under Curve.

## 4 Discussion

To the best of our knowledge, this study is the first to report on the prevalence of anxiety symptoms and its influencing factors in Chinese older adults with cataracts. Based on a nationally representative database, we explored the factors influencing anxiety symptoms in cataract patients in terms of socio-demographic characteristics, lifestyle and health status, and introduced Random Forest(RF) to rank the importance of significant factors. Our findings showed that the prevalence of anxiety symptoms among Chinese older adults with cataracts was 11.06%, which was higher than 7.6% reported by Huang Y et al. (36). The results of a study among Australian older adults with cataracts demonstrated a prevalence of anxiety symptoms of 10.08%, which was significantly higher than 8.3% in normal Australian older adults (37).This may be explained by the findings of Evans, JR et al., that people with cataracts have reduced vision and are more likely to have other symptoms or diseases, that increase the risk of anxiety and depression compared to people with normal vision (38).

Our finding that age has a protective effect on anxiety symptoms in cataract patients is consistent with the results of a longitudinal study exploring the development of depression and anxiety symptoms in older adults with visual impairment (19). Other findings addressing physiological mechanisms in older adults suggest a different view, namely that aging increases the risk of developing anxiety symptoms (39–41). Risk assessment for anxiety symptoms often requires a combination of physiological, psychological and social perspectives. Psychologically, older adults are better able to cope with changing situations because their stress tolerance and self-regulation ability increase with experience. It is not difficult to speculate that the vision problems caused by cataracts may increase anxiety symptoms, but aging may allow for more mature coping mechanisms, which may perhaps further alleviate the development of anxiety symptoms. From a social perspective, compared to younger people, older adults have more satisfying social networks (42) and fewer negative interactions with family members and social network members (43, 44), which can have a positive impact on older adults’ mental health. At the same time, older people have fewer social responsibilities, and their life, work, and marital stresses may be correspondingly lower, which also helps the older adults to avoid negative emotions such as anxiety and depression.

Body Mass Index(BMI) also has a more significant effect on anxiety symptoms in cataract patients. Previous studies have shown that obesity is associated with the development of anxiety symptoms (45). Obesity may contribute to the development of anxiety disorders through a variety of pathways. Psychologically, obesity-induced discrimination and shame (46) as well as reduced quality of life (47) may lead to the development of anxiety symptoms in obese individuals. Physiologically, the range of chronic diseases and dysfunctions caused by obesity may also further increase the risk of developing anxiety symptoms (48, 49). At the same time, anxiety symptoms may also further bring about weight gain by triggering a hypothalamic-axis-driven passive response, whereby an increase in cortisol stimulates cravings for high-calorie foods, further contributing to accumulation of fat (50, 51).In addition to this, it has been shown that the risk of cortical clouding of the lens also increases with a rise in BMI (52),i.e. there is also a link between obesity and the development or exacerbation of cataracts.

Other factors that have a more pronounced effect on the anxiety symptoms of cataract patients include self-reported health(SRH) and economic status. SRH refers to an individual’s self-assessment of his or her health, which is not only a measure of subjective perception of health, but also a reflection of psychological state of the individual, such as the sense of well-being (53). The results of the present study revealed that patients who reported their health as poor were more prone to anxiety. It has been shown that SRH is related to the development of anxiety symptoms, and that there is an correlation between the level of SRH and the severity of anxiety symptoms (54, 55). Self-reported economic status is an important indicator of an individual’s economic status. Previous studies have shown that economic status affects mental health through both material and psychosocial pathways, with a positive correlation between better economic status and lower likelihood of developing psychological disorders (56). More favorable material conditions can reduce the psychological burden of cataract patients due to daily life consumption and the cost of medical care for chronic diseases. At the same time, lower levels of SRH and economic status have also been found to be associated with an increased prevalence of chronic diseases, which may further affect patients’ anxiety symptoms (57, 58).

Fruits consumption habit and sleep duration, two lifestyle-related factors, also demonstrated a significant correlation with anxiety symptoms in cataract patients in the present study. Previous studies have confirmed that fruits contain a variety of substances such as vitamins that are beneficial to mental health (59), suggesting that fruits intake is associated with anxiety symptoms to some extent, which is consistent with the results of the present study. Sleep duration is to some extent an indicator of sleep quality, and the results of the present study revealed that cataract patients with low sleep duration (less than 7h/day) were more likely to experience anxiety symptoms. Evidence from other studies also suggests that poor sleep quality negatively affects the mental health of the population (60), which in turn causes the onset of anxiety symptoms in patients. Therefore, sleep is one of the important influencing factors on anxiety symptoms in cataract patients.

In addition to this, life satisfaction has a more significant effect on anxiety symptoms in cataract patients. Life satisfaction can be seen as a subjective assessment of the quality of life. Many studies have confirmed the negative correlation between quality of life and anxiety symptoms in people (61). This finding applies to cataract patients. In addition, hearing impairment is one of the most important factors influencing anxiety symptoms in cataract patients. Studies have confirmed the association between hearing impairment and psychiatric disorders such as anxiety symptoms, but further research is needed to investigate the direction of causation and other correlations (62). Hearing impairment may cause anxiety symptoms in patients by limiting their social connections in daily life. It has even been found that the dual impairment of hearing and vision exacerbates the occurrence of anxiety symptoms and psychiatric disorders in patients compared with those with visual impairment (63). This is consistent with the finding in the present study that hearing impairment is associated with anxiety symptoms in patients with cataracts, but the specific physiological mechanisms of the effect need to be further investigated.

The results of the study also demonstrated that age was the most important factor affecting anxiety symptoms in older adults with cataracts, and the other factors were, in order of importance, BMI, SRH, economic status, eating fruits or not, sleep duration, life satisfaction, and hearing impairment. Therefore, the primary improvement strategies for anxiety symptoms in older adults with cataracts should focus on the impact of age. This suggests that under the current trend of vigorously exploring nursing strategies for the older adults in the medical industry, it is still necessary to pay attention to the mental health needs of the younger elderly groups. Secondly, for cataract patients with higher or lower BMI, clinicians should provide personalised intervention programmes, including nutritional guidance, sensible diet, and exercise interventions, to help patients improve their weight status and thus reduce anxiety symptoms. Healthcare providers such as hospitals can promote interdisciplinary collaboration among ophthalmologists, dietitians, psychologists and exercise therapists to provide comprehensive weight management and mental health interventions for cataract patients. In addition, there is an urgent need for the public health sector to take action to deepen cataract patients’ knowledge and assessment of their own health status by widely disseminating health knowledge through diversified channels such as community forums, television and radio media and online platforms. Public health education and promotion should emphasise the importance of individual assessment and perception of their own health status, and encourage cataract patients to conduct regular health self-assessment and to detect and report psychological problems in a timely manner. This study calls on the government and the community to pay attention to the economic plight of cataract patients, and to effectively reduce their financial burden by improving health insurance policies, providing medical assistance and financial support. In terms of nutritional guidance, a balanced diet should be advocated, and the intake of foods rich in vitamins and minerals, especially fruits, should be appropriately increased in order to strengthen the body’s resistance. For cataract patients with hearing impairment, hearing screening should be implemented and hearing assistive devices or interventions should be provided to reduce the negative impact of hearing impairment on mental health. In summary, this study suggests that public health departments should consider a comprehensive multilevel intervention strategy to enhance the quality of life and reduce anxiety symptoms in older adults with cataracts.

## 5 Conclusion

Based on the CLHLS database, this study assessed the prevalence of anxiety symptoms and related influencing factors in Chinese older adults with cataracts aged 65 years and older, and ranked the importance of the influencing factors using the random forest model.The results of the study showed that the prevalence of anxiety symptoms among Chinese older adults with cataracts was 11%. Poor economic status, social inactivity, obesity(Body Mass Index>28), poor self-reported health(SRH), sleep duration less than 7h, fair or poor life satisfaction, and hearing impairment were risk factors for anxiety symptoms in cataract patients, and eating fresh fruits and age were protective factors for anxiety symptoms in cataract patients. Age had the greatest effect on the anxiety symptoms of cataract patients, followed by Body Mass Index, SRH, economic status, eating fruits, sleep duration, life satisfaction, and hearing impairment. Based on this, we suggest that healthcare professionals should conduct extensive psychological surveys when treating and caring for older cataract patients, strengthen the monitoring of the above-mentioned influencing indicators, and take psychological as well as pharmacological interventions in a timely manner. Due to the limitations of our use of publicly available databases, future studies should conduct more comprehensive and precise studies on the mental health of cataract patients to verify and consider some factors that may not be adequately considered.

## 6 Limitations

Firstly, the results of this study were based on cross-sectional data, which made it difficult to make causal inference about the etiology. Therefore, we need to further discuss this topic in future studies in conjunction with a longitudinal study design. Second, thesamples included in this study were all older cataract patients, and the effects of these factors on non-cataract patients were not further explored. Future studies may consider setting up a control group of non-cataract patients to further investigate the role of influencing factors in this context. Third, the data in this study were all derived from self-reporting by the respondents, and there may be subjective information biases, which may affect the objectivity and authenticity of the results. Fourth, the data in this study was limited by the content of the public database, and some potentially important influencing factors were not included in the study (e.g., taking medications, healthcare services, cataract surgery, other types of chronic diseases, etc.). What’s more, the database did not further collect diagnostic histories of cataracts and anxiety symptoms, which should be considered as part of a more comprehensive design for investigation in future studies. Fifth, there may also be other interferences related to eye diseases in the screening process of cataract patients, and future studies should consider further eliminating these interferences. Finally, due to the specificity of the cataract patient population, the national representativeness of the sample used in our study may have been somewhat insufficient, and thus all the conclusions in this paper deserve further justification in future studies with large samples.

## Data Availability

Publicly available datasets were analyzed in this study. This data can be found here: https://opendata.pku.edu.cn/dataset.xhtml?persistentId=doi:10.18170/DVN/WBO7LK, Chinese Longitudinal Healthy Longevity Survey.
