# Supplementary material for: Prevalence and main factors influencing anxiety symptoms in Chinese older adults with cataracts: a national cross-sectional survey
Source: Front Psychiatry. 2025 Jun 2;16:1534863. doi: 10.3389/fpsyt.2025.1534863 (PMC12169137; doi:10.3389/fpsyt.2025.1534863)
Supplement: Supplementary file 1 [file Table1.docx]

**Appendix 1 Measurements and Classification Criteria of Covariates.**

| **Variables** | **Questionnaire Items** | **Classification Criteria** |
| --- | --- | --- |
| Age | What’s your date of birth? | Continuous variable |
| Gender | What’s your gender? | Female=0; Male=1 |
| Education | How many years of education did you have? | 0=0; 0-6=1;>6 =2 |
| Marital status | What is your marital status? | Not married=0; Married=1 |
| Place of residence | Was your address in the urban or rural? | Rural=1; Urban=0 |
| Economic status | How do you rate your economic status compared with other local people? | Good=0; Fair=1; Bad=2 |
| BMI^a^ | How much do you weight; What’s your height? | 18.5-24=0;<18.5=1;24-28=2;>28=3 |
| Abdominal obesity^b^ | What is your waist circumference? | No=0; Yes=1 |
| Hearing impairment | Do you have hearing impairment? | No=0; Yes=1 |
| Hypertension | Have you been diagnosed with Hypertension by a doctor? | No=0; Yes=1 |
| Diabetes | Have you been diagnosed with Diabetes or high blood sugar by a doctor? | No=0; Yes=1 |
| Cardiopathy | Have you been diagnosed with heart disease by a doctor? | No=0; Yes=1 |
| SRH | How do you feel about your health right now? | Good=0; Fair=1; Bad=2 |
| ADL disability^c^ | Whether you need help with bathing, getting dressed, toilet and urinal, indoor activities and eating; whether you can control your bowel? | No=0; Yes=1 |
| Eating fruits | Do you eat fresh fruits ? | Never or rarely=0;Occasionally or quite often=1; Almost everyday =2 |
| Eating vegetables | Do you eat fresh vegetables ? | Never or rarely=0;Occasionally or quite often=1; Almost everyday =2 |
| Taste | What's your taste? | Not light=0; Light=1 |
| Sleep duration | How many hours do you typically sleep a day right now? | 7-9=0;<7=1;>9=2 |
| Smoking | Have you ever smoked? | No=0; Yes=1 |
| Drinking | Did you ever drink alcoholic beverages in the past? | No=0; Yes=1 |
| Exercise | Do you exercise regularly now? | No=0; Yes=1 |
| Physical labour | Do you now participate in manual labour? | No=0; Yes=1 |
| Social activity | Do you participate in social activities? | No=0; Yes=1 |
| Life satisfaction | How do you feel about your life right now? | Good=0; Fair=1; Bad=2 |

Note: BMI, Body Mass Index; SRH, Self-Reported Health; ADL, Activities of Daily Living;

a: BMI= Weight/The square of the height(1);

b: Abdominal obesity: Male waist circumference>=90cm; female waist circumference>=85cm(2);

c: ADL disability: ADL disability was measured using the Katz Index, which consists of six items: bathing, dressing, toileting, indoor transferring, eating, and control of defecation and urination. Participants' responses to each question were categorized into three levels: 1. without assistance; 2. one part assistance; 3. more than one part assistance. Participants who chose 2 or 3 were considered to have a disability for that item. Whenever one of the six items was assessed as having a disability, the participant was recognized as having an ADL disability(3).

**Reference**

1. Chen C, Lu FC. The Guidelines for Prevention and Control of Overweight and Obesity in Chinese Adults. *Biomedical and environmental sciences : BES* (2004) 17 Suppl:1-36. Epub 2005/04/06.

2. Zhang L, Wang Z, Wang X, Chen Z, Shao L, Tian Y, et al. Prevalence of Abdominal Obesity in China: Results from a Cross‐Sectional Study of Nearly Half a Million Participants. *Obesity* (2019) 27(11):1898-905. doi: 10.1002/oby.22620.

3. Katz S, Ford AB, Moskowitz RW, Jackson BA, Jaffe MW. Studies of Illness in the Aged. The Index of Adl: A Standardized Measure of Biological and Psychosocial Function. *Jama* (1963) 185:914-9. Epub 1963/09/21. doi: 10.1001/jama.1963.03060120024016.
